# Simultaneous Lattice Engineering and Defect Control via Cadmium Incorporation for High‐Performance Inorganic Perovskite Solar Cells

**DOI:** 10.1002/advs.202204486

**Published:** 2022-11-07

**Authors:** Tianfei Xu, Wanchun Xiang, Dominik J. Kubicki, Yali Liu, Wolfgang Tress, Shengzhong Liu

**Affiliations:** ^1^ Key Laboratory of Applied Surface and Colloid Chemistry Ministry of Education Shaanxi Key Laboratory for Advanced Energy Devices Shaanxi Engineering Lab for Advanced Energy Technology School of Materials Science and Engineering Shaanxi Normal University Xi'an 710119 China; ^2^ Department of Physics University of Warwick Coventry CV4 7AL UK; ^3^ Institute of Computational Physics Zurich University of Applied Sciences Wildbachstr. 21 Winterthur 8401 Switzerland; ^4^ Dalian National Laboratory for Clean Energy iChEM Dalian Institute of Chemical Physics Chinese Academy of Sciences Dalian 116023 China

**Keywords:** doping, inorganic perovskites, perovskite solar cells, power conversion efficiency, stability

## Abstract

Doping of all‐inorganic lead halide perovskites to enhance their photovoltaic performance and stability has been reported to be effective. Up to now most studies have focused on the doping of elements in to the perovskite lattice. However, most of them cannot be doped into the perovskite lattice and the roles of these dopants are still controversial. Herein,the authors introduce CdI_2_ as an additive into CsPbI_3−*x*_Br_*x*_ and use it as active layer to fabricate high‐performance inorganic perovskite solar cells (PSCs). Cd with a smaller radius than Pb can partially substitute Pb in the perovskite lattice by up to 2 mol%. Meanwhile, the remaining Cd stays on the surface and grain boundaries (GB) of the perovskite film in the form of Cs_2_CdI_4−*x*_Br_−*x*_, which is found to reduce non‐radiative recombination. These effects result in prolonged charge carrier lifetime, suppressed defect formation, decreased GBs, and an upward shift of energybands in the Cd‐containing film. A champion efficiency of 20.8% is achieved for Cd‐incorporated PSCs, together with improved device ambient stability. This work highlights the importance of simultaneous lattice engineering, defectcontrol and atomic‐level characterization in achieving high‐performance inorganic PSCs with well‐defined structure‐property relationships.

## Graphical abstract

The incorporation of CdI_2_ results in simultaneous lattice engineering and defect control of inorganic perovskite, which is found to reduce nonradiative recombination and prolonged charge carrier lifetime. As a result, the power conversion efficiency of inorganic perovskite solar cells is significantly improved from 19.5% to 20.8%, together with improved device ambient stability.

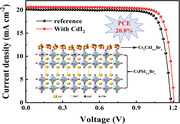

## 1 Introduction

Metal halide perovskite materials, due to their excellent optoelectronic properties, such as tunable bandgap, high absorption coefficient, and relatively large diffusion length for charge carriers, are very attractive for the application in photovoltaic technologies.^[^ ^1^, ^2^ ^]^ The certified power conversion efficiency (PCE) of n‐i‐p solar cells using organic–inorganic hybrid perovskite as light absorber has achieved 25.7%,^[^ ^3^ ^]^ which is very close to silicon solar cells. Nevertheless, the organic components in perovskites generally suffer from thermal instability,^[^ ^4^, ^5^ ^]^ which is a potential threat for the long‐term operational stability of perovskite solar cells (PSCs). The replacement of the organic components by inorganic ions (Cs^+^, Rb^+^) thus can fully solve the thermal stability issue.^[^ ^6^, ^7^, ^8^ ^]^

CsPbI_3_ is a highly relevant representative of inorganic halide perovskite for solar cells as it possesses the ideal bandgap of 1.73 eV for the top cell in a tandem configuration with a silicon bottom cell.^[^ ^9^, ^10^ ^]^ Owing to the soft‐ionic nature of polycrystalline perovskites, defects are easily formed on the surface and grain boundaries (GBs) of the films by commonly used solution‐processing methods.^[^ ^11^, ^12^ ^]^ In particular, point defects such as halide anion vacancies are prone to form due to their low formation energies resulting from the weak bonding within the perovskite lattice.^[^ ^13^ ^]^ For example, the high annealing temperature required for perovskite phase formation accelerates the escape of iodide, leaving abundant halide vacancies and yielding iodine‐deficient composition.^[^ ^14^ ^]^ Although halide vacancies are normally shallow electronic traps, they still can either capture free charge carries to restrain their diffusion lengths, or act as recombination centers to terminate lifetime of charge carriers, thus narrowing the quasi‐Fermi‐level splitting within perovskite and deteriorating the maximum open circuit voltage (*V*_OC_).^[^ ^15^, ^16^, ^17^ ^]^ More importantly, under operational conditions, the diffusion of these halide and other ionic vacancies into the crystallites can promote the migration of anions to the surfaces and GBs, which then produces additional halide vacancies and leads to an irreversible decomposition reactions at these locations.^[^ ^18^ ^]^ Moreover, vacancy defects have high affinity toward water and oxygen molecules, accelerating perovskite degradation via a vacancy assisted decomposition mechanism.^[^ ^8^, ^19^, ^20^ ^]^ Therefore, control and mitigation of halide anion vacancies and suppression of the newly formed defects through effective defect engineering or chemical modulation has become an important research direction towards more efficient and stable inorganic PSCs.

A number of strategies have been proposed to alleviate the halide vacancies within perovskites, including materials’ compositional engineering,^[^ ^21^, ^22^ ^]^ dimensionality engineering,^[^ ^23^, ^24^ ^]^ or intermediate phase engineering,^[^ ^25^, ^26^ ^]^ in ways of either modulating lattice composition or improving film quality. A straightforward way is to introduce additional iodide source to compensate for the loss of the stoichiometric iodide from the perovskite.^[^ ^15^, ^27^ ^]^ However, the intrinsic issue of the low bonding strength of iodide within the lead halide octahedra still exists. Alternatively, depositing an organic passivation layer to form acid‐base adducts on the surface and GBs also suppresses the vacancy defect density, as has been widely reported in the literature.^[^ ^13^, ^28^, ^29^, ^30^ ^]^ However, the introduction of the extra organic layer adds the risk of decreases stability of the solar cells. Yet another strategy is to induce the formation of a 2D structure on the surface and GBs by using large‐size organic moiety to prevent moisture infiltration. The downside of this approach is that the low‐dimensional materials generally exhibit inferior charge transfer properties as the charge carrier conduction is constrained along the inorganic octahedral planes.^[^ ^31^, ^32^ ^]^

Compositional engineering is the most prevalent method to modulate the perovskite crystal lattice and suppress defect formation, as it is straightforward and does not involve additional passivation layers, and thus beneficial for reducing the production costs. If the dopant is not incorporated into the perovskite lattice, it can form separate phases and may modify the surface and GBs of the perovskite by defect passivation or manipulation of grain growth.^[^ ^33^, ^34^ ^]^ If the dopant is incorporated into the perovskite lattice by replacing one of its original components, it can substantially increase the entropy of the perovskite lattice and enhance thermal stability.^[^ ^35^ ^]^ Therefore, in evaluating new dopants, it is essential to identify their speciation, i.e., distribution within the different phases, and relate this information to their function, thereby establishing a structure‐function relationship. Solid‐state nuclear magnetic resonance (NMR) has played a key role in determining the speciation of dopants in metal halide perovskites.^[^ ^36^ ^]^ Owing to its relatively low sensitivity compared to other spectroscopies, it has been essential to develop synthetic strategies yielding large quantities of halide perovskites (on the order of 10–500 mg) of similar quality to those made by spin coating to make solid‐state NMR measurements viable. Mechanochemistry emerged as a solution to this problem because it yields high quality halide perovskites which, in terms of structure, are largely identical to those made by solution processing.^[^ ^36^, ^37^ ^]^ This synthetic protocol also has numerous other advantages: shorter reaction times, nearly 100% atom economy, elimination of solubility issues of inorganic precursors, and does not produce any liquid waste.^[^ ^37^ ^]^ While the long‐range and local structure of halide perovskites made by mechanosynthesis and solution routes has been shown to be essentially indistinguishable across a number of materials,^[^ ^36^ ^]^ perovskites made by mechanosynthesis have been also used, after dissolution, in the fabrication of optoelectronics devices and showed improved performance over their purely solution‐processed counterparts.^[^ ^37^ ^]^ Here, we use materials made by mechanosynthesis to study the speciation of cadmium using ^113^Cd solid‐state NMR. Previous studies have revealed that substitution of halide or lead site can noticeably alter the tolerance factor of the lattice, as well as the frontier orbital distribution of the materials.^[^ ^35^, ^38^ ^]^ For example, Saidaminov et al. introduced small ions of cadmium and chloride into triple‐cation perovskite to suppress the formation of halide vacancies via lattice strain relaxation.^[^ ^39^ ^]^ Kubicki et al. have later shown that cadmium does not incorporate into this material and instead segregates into cadmium‐rich phases, which are the likely cause of the observed effects.^[^ ^40^ ^]^ We have found that partial replacement of lead by europium, which has a smaller ionic radius, increases the ambient stability of the CsPbI_2_Br perovskite.^[^ ^41^ ^]^ In another study, we found that barium cannot incorporate into the inorganic perovskite lattice, but rather that its effect is to form a robust passivation layer around GBs, which suppresses nonradiative charge recombination.^[^ ^42^ ^]^

In this work, we adopted CdI_2_ as an additive in perovskite precursor solutions to mitigate halide vacancies within iodide‐rich inorganic perovskite films. The ionic radius of the cadmium ion (95 pm for hexacoordinate Cd^2+^) is smaller than that of Pb (119 pm), so that a reduced perovskite lattice distortion is expected upon substitution of Pb^2+^ with Cd^2+^. A previous solid‐state NMR study found that Cd^2+^ has the capacity to incorporate into the all‐inorganic CsPbBr_3_, which inspired us to use this dopant in a mixed‐halide composition.^[^ ^40^ ^]^ We found that Cd can be incorporated into CsPbI_3−_ *_x_* Br*_x_* (0 < *x* ≤ 0.2) perovskite lattice by up to 2 mol% relative to Pb. Simultaneously, the excess Cd leads to in situ formation of cadmium‐rich phases that accumulate at the surface of the perovskite film and GBs, which reduces nonradiative recombination. The addition of CdI_2_ leads to significantly increased perovskite grain sizes. Furthermore, we observed that the iodides from CdI_2_ participate in the suppression of halide vacancies by providing a supplementary iodide source. These combined effects enable substantial enhancement of photovoltaic parameters of inorganic PSCs and yield a champion PCE of 20.8% under 100 mW cm^−2^ irradiation, which is among the highest reported PCEs for inorganic PSCs. Finally, the addition of CdI_2_ enhances the ambient stability of unencapsulated CsPbI_3−_ *_x_* Br*_x_* PSCs by leading to retention of 90% of the initial efficiency after 600 h.

## 2 Results and Discussion

### 2.1 Photovoltaic Performance

**Figure 1:**
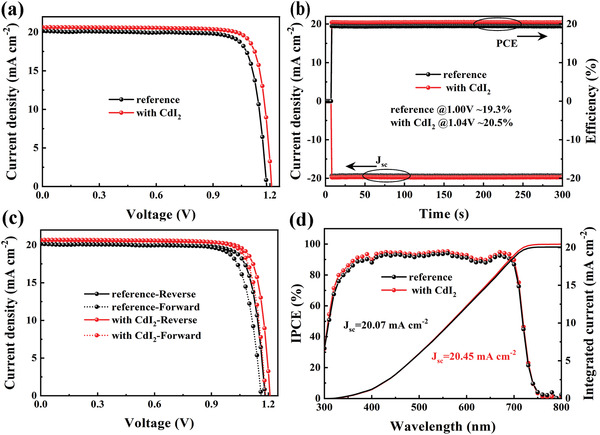
Photovoltaic performance of CsPbI_3−_ *_x_* Br*_x_* based inorganic PSCs without and with 8 mol% CdI_2_ addition. a) *J–V* performance, b) SPO; c) hysteresis, and d) IPCE and integrated *J*_SC_.

A range of different Cd^2+^ amounts was added in the preparation of the cesium inorganic perovskite films and they were used to fabricate PSCs with the configuration of fluorine‐doped tin oxide (FTO)/*c*‐TiO_2_/perovskite/2,2′,7,7′‐tetrakis(N,N‐di‐p‐methoxyphenylamine)‐9,9‐spirobifluorene (spiro‐OMeTAD)/Au. We used dimethylamine lead triiodide (DMAPbI_3_) as one of the perovskite precursors because DMA^+^ facilitates the formation of phase stable inorganic perovskite via intermediate phase engineering. The *J–V* performance of these PSCs was evaluated, and the best results for each CdI_2_ amount are shown in Figure S1 (Supporting Information) and **Table** **1**. The reference device without Cd‐doping exhibits a *V*_OC_ of 1.18 V, a short‐circuit current (*J*_SC_) of 20.14 mA cm^−2^, a fill factor (FF) of 81.93%, and a PCE of 19.5%, similar to those reported in previous work.^[^ ^13^ ^]^ Upon addition of 8 mol% CdI_2_, the PSCs demonstrate substantially higher FFs of up to 83.2%, together with improved *J*_SC_ and *V*_OC_ of 20.64 mA cm^−2^ and 1.21 V, yielding a champion PCE of 20.8%, which is among the highest reported values for inorganic perovskite‐based solar cells (**Figure** **1**a).^[^ ^13^, ^43^ ^]^ The corresponding stabilized power output (SPO) of the champion cell measured at maximum power point (MPP) shows a value of 20.5%, with a current density of 19.7 mA cm^−2^ in a testing period of 5 min, signifying a stable power output of the Cd^2+^‐doped devices (Figure 1b). The notorious hysteresis effect of the champion solar cells after Cd‐doping is clearly improved, with a hysteresis index decreasing from 3.94% without Cd^2+^ doping to 1.93% with Cd^2+^ doping (Figure 1c, see detailed data in Table S3, Supporting Information). The incident photon‐to‐current conversion efficiency (IPCE) curve of the champion cell is shown in Figure 1d, with an external quantum efficiency (EQE) plateau of over 90% in a wavelength range between 400 and 710 nm. A further increase in the CdI_2_ concentration leads to a deterioration of all three parameters. These results clearly show that Cd^2+^ doping has a remarkably positive impact on photovoltaic performance of CsPbI_3−_ *_x_* Br*_x_* PSCs. The statistics of photovoltaic performance with different Cd‐containing PSCs are shown in Figure S2 (Supporting Information). For comparison, we also fabricated inorganic PSCs using PbI_2_ as an additive instead of CdI_2_, whose *J–V* performance is shown in Figure S3 (Supporting Information). The PbI_2_‐doped PSCs exhibit lower *V*_OC_ as well as PCE compared to CdI_2_‐doped devices, although slightly higher PCE than reference devices, confirming that it is the Cd^2+^ cation that is mainly responsible for the large improvement of *J–V* performance. In the following sections we investigate the role of Cd^2+^ in producing this enhancement.

**Table 1:** *J–V* parameters of PSCs based on CsPbI_3−_ *_x_* Br*_x_* with different amounts of CdI_2_. These parameters were obtained from a reverse scan direction using the best performing devices for each concentration and were recorded under 100 mA cm^−2^ illumination with an aperture area of 0.09 cm^2^

CdI_2_ amount [mol%]	*V*_OC_ [V]	*J*_SC_ [mA cm^−2^]	FF [%]	PCE [%]
0	1.18	20.14	81.9	19.5
3	1.20	20.17	81.7	19.9
8	1.21	20.64	83.2	20.8
13	1.17	20.01	80.4	18.9

### 2.2 Material Composition and Cd^2+^ Distribution within Perovskite Films

**Figure 2:**
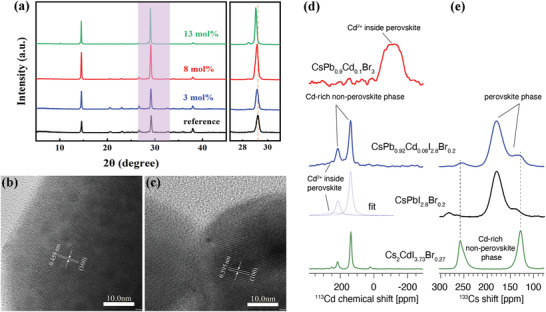
Perovskite film characterization. a) XRD patterns of perovskite films with and without CdI_2_ doping; lattice parameter measurement by TEM on a sample b) without Cd^2+^ and c) with Cd^2+^, the scale bar is 10 nm; solid‐state NMR characterization of materials made by solid‐state mechanosynthesis: d) ^113^Cd and e) ^133^Cs MAS NMR spectra. The CsPb_0.9_Cd_0.1_Br_3_ spectrum in panel (d) is adapted with permission from ref. [40], American Chemical Society.

To assess the long‐range structure of the perovskite films, we conducted X‐ray diffraction (XRD) measurements and collected the patterns in **Figure** **2**a. All perovskite films exhibit characteristic peaks located at 14.6° and 29.2°, corresponding to the (100) and (200) planes, respectively. For all Cd^2+^‐doped films, an additional peak appears at 28.4°, which we attribute to a Cd^2+^‐rich phase. The intensity of this peak increases with the increasing amount of CdI_2_, suggesting that phase segregation to a Cd^2+^‐rich phase occurs. The intensity of the perovskite peaks in the Cd^2+^‐doped films is considerably stronger than those of the reference film, implying improved crystallinity of the perovskite as a result of doping. A scrutiny of the XRD patterns reveals a small shift of the perovskite peaks toward lower angles (29.22° for reference, 29.06° for 13 mol%), indicating lattice expansion. The transmission electron microscope (TEM) images corroborate this conclusion in that they show the lattice spacing increasing from 0.458 to 0.595 nm upon 8 mol% CdI_2_ addition (Figure 2b,c). Since the ionic radius of hexacoordinate Cd^2+^ is smaller than that of Pb^2+^, the replacement of Pb^2+^ with Cd^2+^ in the structure should lead, on average, to a smaller lattice parameter, and therefore induce a shift towards higher angles. We hypothesize that the experimentally observed XRD peak shift toward lower angles may be caused by: 1) Cd^2+^ accumulation at the interstitial lattice sites, which may release lattice strain leading to an increase in Pb—I—Pb angles;^[^ ^44^ ^]^ or 2) change in the iodide‐to‐bromide ratio, with a higher iodide concentration leading to lattice expansion. This result shows that using XRD alone it is difficult to unambiguously determine whether Cd^2+^ is incorporated into the perovskite lattice because the shift induced by Cd^2+^ incorporation may be obscured by the shift in the opposite direction caused by an increase in the iodide concentration in the lattice as a result of phase segregation.

To understand the speciation of cadmium in this material, we carried out solid‐state magic angle spinning (MAS) NMR measurements. Solid‐state MAS NMR is a versatile strategy for studying the speciation of dopants and phase segregation phenomena in halide perovskites. A recent ^113^Cd MAS NMR study has shown that Cd^2+^ does not incorporate into the structure of hybrid materials based on methylammonium (MA) and formamidinium (FA), but it does so into the all‐inorganic CsPbBr_3_.^[^ ^40^ ^]^ The materials used for the NMR study were prepared using solid‐state mechanosynthesis to provide the large quantity of sample needed for studying the highly dilute Cd^2+^ dopant. The signature of Cd^2+^ inside the perovskite lattice of CsPbBr_3_ is a very broad signal spanning the region between 0 and 200 ppm (Figure 2d).^[^ ^40^ ^]^ On the other hand, phase segregation into Cd^2+^‐rich nonperovskite phases leads to the appearance of a distinct pattern of sharp peaks corresponding to unique local environments with different halide composition. In the A_2_CdI_4−_ *_x_* Br*_x_* (A = MA, FA, Cs) materials, the Cd site is tetrahedrally coordinated and therefore the local environments resolved in their ^113^Cd spectra correspond to the CdBr_4_, CdIBr_3_, CdI_2_Br_2_, and CdI_3_Br tetrahedra.^[^ ^40^ ^]^ The situation we observe in the material corresponding to 8 mol% CdI_2_ doping (prepared using the nominal formula CsPb_0.92_Cd_0.08_I_2.8_Br_0.2_) is a combination of these two scenarios (Figure 2d). There are two distinct narrow peaks (at 140 ppm, fwhm 24 ppm, and at 214 ppm, fwhm 30 ppm), which correspond to a Cd^2+^‐rich nonperovskite phase that accounts for 78% of Cd^2+^ in the material. The identity of this phase is confirmed by preparing a reference Cs_2_CdI_4−_ *_x_* Br*_x_* phase in which the I:Br ratio corresponds to that used in the perovskite composition (2.8:0.2), i.e., Cs_2_CdI_3.73_Br_0.27_. This reference phase yields a spectrum which perfectly matches the narrow components seen in Cd^2+^‐doped perovskite. The deconvolution of the ^113^Cd spectrum of CsPb_0.92_Cd_0.08_I_2.8_Br_0.2_ also yields a very broad component (241 ppm, fwhm 164 ppm), which qualitatively resembles that of Cd^2+^ incorporated into CsPbBr_3_, and we therefore attribute it to Cd^2+^ incorporated into the perovskite lattice in the mixed‐halide composition. This component accounts for 22% of Cd^2+^ in the composition, which for the nominal doping level of 8 mol% translates to ≈2 mol% Cd^2+^ incorporated into the perovskite. A spectrum recorded with a longer recycle delay (60 s, Figure S4, Supporting Information) does not show substantial changes in the relative intensities of the two species which allows us to conclude that the spectrum recorded with a shorter recycle delay (2.5 s, Figure 2d) is largely quantitative. This result allows us to determine the effective stoichiometry of the perovskite phase as approximately CsPb_0.98_Cd_0.02_I_2.8_Br_0.2_, with the remainder of the material consisting of a Cd^2+^‐rich nonperovskite phase with the composition of approximately Cs_2_CdI_3.73_Br_0.27_. Based on the comparison of CsPb_0.9_Cd_0.1_Br_3_, which features Cd^2+^ mostly incorporated into the perovskite phase, and of our nominal composition CsPb_0.92_Cd_0.08_I_2.8_Br_0.2_, where only 22% of Cd^2+^ is incorporated into the perovskite phase, we conclude that the addition of iodide reduces the capacity of Cd^2+^ to incorporate into CsPbI_3−_ *_x_* Br*_x_* perovskite structures. We also recorded the corresponding ^133^Cs MAS NMR data (Figure 2e). The comparison of reference CsPbI_2.8_Br_0.2_ and CsPb_0.92_Cd_0.08_I_2.8_Br_0.2_ reveals a new peak at ≈255 ppm and a shoulder at ≈130 ppm in the Cd^2+^‐doped material, which match perfectly the ^133^Cs spectrum of the reference Cd‐rich nonperovskite Cs_2_CdI_3.73_Br_0.27_ phase. The most intense signal corresponding to the perovskite phase is substantially broadened (fwhm 28 ppm) compared to single‐halide perovskites (e.g., fwhm of 4 ppm for CsPbBr_3_) owing to halide disorder,^[^ ^45^ ^]^ that is the presence of a distribution of largely unresolved local cesium environments with different halide compositions. Taken together, the solid‐state NMR results show that at 8 mol% Cd^2+^ doping level, cadmium segregates between the perovskite phase (22%) and a Cd^2+^‐rich nonperovskite phase (78%). In the context of the NMR data, the most plausible explanation for the lattice expansion observed in XRD is that the addition of CdI_2_ leads to partial incorporation of both Cd^2+^ and I^−^ into the perovskite lattice, with the effect of iodide‐induced expansion overbalancing the cadmium‐induced contraction.

**Figure 3:**
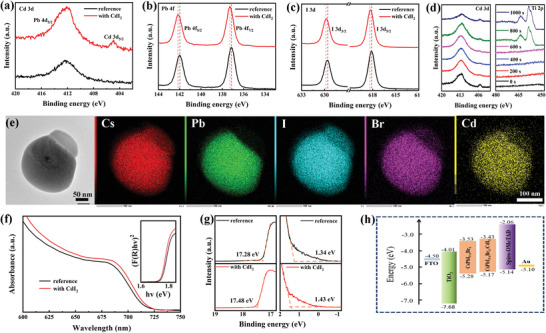
XPS characterization of perovskite films with and without Cd‐doping: a) Cd 3d, b) Pb 4f, and c) I 3d; d) depth‐profile XPS for Cd 3d and Ti 2p. The perovskite film was deposited on TiO_2_ substrate; e) EDS from HAADF‐TEM; f) UV–vis (inset: Tauc plot); g) UPS characterization; h) energy diagram of PSC. The values are relative to vacuum.

We next characterized the films by X‐ray photoelectron spectroscopy (XPS) to further corroborate the changes to local chemical environments induced by 8 mol% CdI_2_ doping. A survey XPS spectrum is shown in Figure S5 (Supporting Information). As shown in **Figure** **3**a, a small peak appears at 404.9 eV in the Cd^2+^‐doped film that we attribute to the Cd 3d signal, which is absent in the reference CsPbI_3−_ *_x_* Br*_x_* sample. The peaks of Pb 4f appearing at 137.1 and 142.0 eV shift to 137.3 and 142.2 eV, respectively, upon CdI_2_ doping (Figure 3b). A similar effect is observed for the I 3d peaks (shift from 618.14 and 629.63 eV, to 618.31 and 629.80 eV, respectively, Figure 3c). These XPS peak shifts indicate that CdI_2_ doping leads to a change in binding energies of the elements constituting the [PbX_6_]4^−^ (X represents halide) octahedra, which is caused by the incorporation of Cd^2+^ into the perovskite structure and a slight change in the I‐to‐Br ratio, as elucidated by XRD and solid‐state NMR. The depth profile XPS analysis from Figure 3d suggests that Cd^2+^ is distributed both within the perovskite film and on the surface of perovskite grains, where the Cd^2+^ signals from the surface are stronger than those from the bulk. It indicates that the majority of Cd^2+^ is distributed on the surface of perovskite film/grains. We also used high‐angle annular dark field scanning transmission electron microscope (HAADF‐STEM) imaging and energy‐dispersive X‐ray spectroscopy (EDS) mapping to probe the Cd^2+^ distribution in the material with 8 mol% CdI_2_ addition. A low‐magnification TEM image is presented in Figure S6 (Supporting Information). Figure 3e shows EDS maps of one of the grains, which shows homogeneous distribution of all the elements, including Cd, in the perovskite grain, corroborating the incorporation of Cd^2+^ into the perovskite structure. In addition, the capping layer on the upper right side consists of all the elements except Pb. This observation suggest that it corresponds to a Cd‐rich phase, in perfect agreement with the solid‐state NMR data which showed that the majority of Cd^2+^ is in the form of Cs_2_CdI_4−_ *_x_* Br*_x_*, with *x* ≈ 0.3, segregated from the parent Pb‐based perovskite.

The introduction of CdI_2_ appreciably increases the absorbance of the corresponding films compared to CsPbI_3−_ *_x_* Br*_x_* reference film (Figure 3f), probably due to the improvement in the film morphology, as discussed later. The Tauc plot indicates a small narrowing of the bandgap upon CdI_2_ doping, most likely owing to the incorporation of extra iodide. The ultraviolet photoelectron spectroscopy (UPS) analysis in Figure 3g also exhibits a small upward shift of the VB level (detailed data are shown in Table S4, Supporting Information), which may facilitate hole transfer at the interface. Accordingly, the integrated energy diagram is summarized in Figure 3h.

### 2.3 Crystallization and Film Morphology

**Figure 4:**
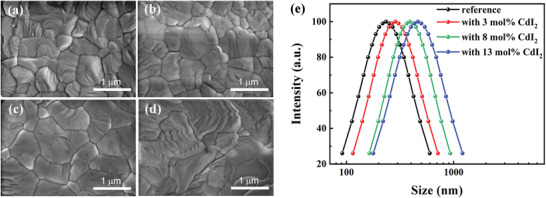
Top‐view SEM images of inorganic perovskite films with different amounts of Cd‐doping: a) reference, b) 3 mol%, c) 8 mol%, and d) 13 mol%, the scale bar is 1 µm; e) size distribution of perovskite–solvent complex in precursor solution with different amounts of CdI_2_.

In the next step, we tried to understand how the introduction of CdI_2_ affects the nucleation and grain growth kinetics of the cesium lead halide inorganic perovskite. We investigated the evolution of the perovskite film morphology with increasing concentration of CdI_2_ doping using top‐view scanning electronic microscope (SEM) (**Figure** **4**a–d). The reference perovskite film exhibits densely packed grains with an average grain size of around 600 nm, featuring a full coverage of the substrate without pinholes, which is crucial to avoid shunt paths between charge transport layers and the associated short‐circuit. With the continuously increasing Cd concentration in the films, we observe that the perovskite grains grow significantly larger, with an average size on the micrometer scale. It has been widely recognized that lattice defects preferentially exist along the GBs due to the lower defect formation energy (DFE) at these sites. Larger grain size means reduced total GB surface area, and consequently suppressed defect density and nonradiative charge recombination.^[^ ^46^, ^47^ ^]^ Higher Cd concentration (13 mol%) results in wrinkles in the film, implying a rougher surface and lower film quality. We also observe that the corresponding material with the same amount of PbI_2_ incorporation does not show obvious morphological variation compared to the reference film (Figure S8, Supporting Information), confirming that Cd cations contribute to the substantial improvement of the film morphology. In addition, perovskite films with Cd‐doping also show a decrease in roughness of the root mean square (RMS) value from 11.3 nm (reference) to 8.15 nm (8 mol%), as is shown by atomic force microscope (AFM) (Figure S9, Supporting Information), confirming the beneficial effect of Cd‐doping for obtaining high‐quality inorganic perovskite films.

To get insight as to why Cd‐doping leads to the change of film morphology, we conducted dynamic light scattering (DLS) characterization on the perovskite precursor solutions with and without CdI_2_, using the latter as a reference. Figure 4e shows that the mean diameter of perovskite–solvent complexes is around 200 nm, while Cd‐containing perovskite precursor solutions exhibit substantially larger colloids than the Cd‐free solution, with higher concentration of Cd corresponding to larger radii. This result implies that the addition of CdI_2_ has a pronounced impact on the nucleation dynamics of perovskite grains, which may be attributed to the strong electrostatic interaction between the Cd^2+^ cation and [PbX_6_]4^−^ cages due to the smaller ionic radius of Cd^2+^ compared to Pb^2+^.^[^ ^27^ ^]^ We hypothesize that the addition of CdI_2_ may reduce the number of nucleation sites, resulting in a reduced nucleation rate during perovskite formation. Slower nucleation rates in perovskite films in the presence of CdI_2_ result in a larger grain size in the perovskite film, provided the growth rate remains unchanged.^[^ ^48^ ^]^ Higher CdI_2_ concentrations in the precursor solution leads to a more pronounced change of the chemical environment of the complex, as is displayed from the variation of ultraviolet–visible (UV–vis) spectra in Figure S10 (Supporting Information), which may possess a negative impact on the film growth, as is observed in SEM characterization.^[^ ^49^, ^50^, ^51^ ^]^ Moreover, we monitored the film crystallization process during annealing by in situ XRD (Figure S11, Supporting Information). The as‐prepared reference film shows a noticeable PbI_2_ peak at 11.92°, which shifts to 11.88° in the as‐prepared Cd‐doped film. None of the as‐prepared films contains the perovskite phase. The slight PbI_2_ peak shift indicates a chemical interaction between Cd cations and PbI_2_, which may influence the crystallization process. Solid‐state NMR has previously shown that PbI_2_ and CdI_2_ can form solid solutions of the form Pb_1−_ *_x_* Cd*_x_* I_2_.^[^ ^40^ ^]^ As annealing progresses, the PbI_2_ gradually disappears and the perovskite phase emerges. The continuous shift of the perovskite peaks toward higher angles indicates the replacement of DMA^+^ by cesium during perovskite formation via intermediate phase engineering.^[^ ^25^, ^26^ ^]^

### 2.4 Suppression of Defects in Thin Films

**Figure 5:**
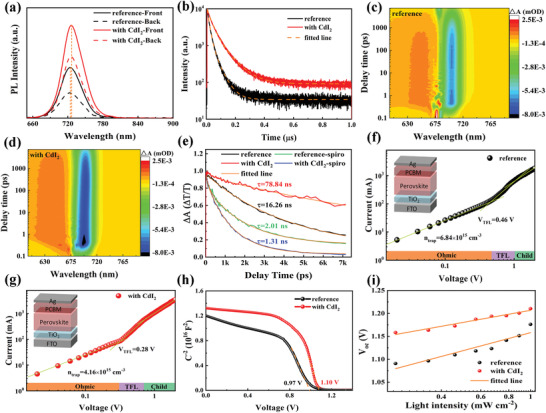
Probing the passivation capacity of the CdI_2_ additive. a) PL spectra; b) TRPL spectra; 2D contour plot of TAS of the photoinduced absorption (Δ*A*) as a function of wavelength and delay time for the c) reference and d) CdI_2_‐doped film; e) dynamic evolution of the bleaching recovery; f,g) defect density measured by SCLC; h) *C*–*V* measurement; i) *V*_OC_ versus light intensity.

We have previously identified that the main defect type for inorganic halide perovskite is the halide vacancy. Specifically, for iodide‐rich perovskite compositions, the iodide vacancy defect is more prone to form on the surface and GBs (DFE of −1.21 eV) than in the bulk (DFE of 0.44 eV), where lower energies indicate easier formation.^[^ ^13^ ^]^ Here, using density functional theory (DFT), we found that the DFE of an iodide vacancy on the (100) surface with PbI_2_ termination substantially increases to 1.58 eV with Cd‐doping (Figure S12, Supporting Information). This result suggests that the formation of halide vacancies on the surface or GBs of perovskite is expected to be greatly inhibited by CdI_2_ doping. We hypothesize that this is most likely because the Cd^2+^ cations bind more strongly to the halides through electrostatic interactions compared Pb^2+^ owing to the smaller ionic radius of Cd^2+^ than Pb^2+^. We tested this hypothesis by carrying out photoluminescence (PL) measurements to probe the charge recombination pathways in the films (**Figure** **5**a). The PL intensity measured using 510 nm excitation through the perovskite side (traces labeled as “Front”) increased for the CdI_2_ doped film by a factor of 40% compared to the reference film. When the laser was incident through the glass substrate (traces labeled as “Back”), the PL intensity of the perovskite film doped with CdI_2_ increased by 58%. This result confirms that defects in the film have been successfully suppressed by CdI_2_, which we attribute to the additive being well distributed throughout the film. In addition, we observed a small redshift of the PL peak (2.5 nm) for the CdI_2_‐doped film, which is also visible in the UV–vis spectra as a small redshift of the absorption onset. We attribute this shift mainly to a change in the band structure induced by the incorporation of additional iodide into the perovskite lattice, as evidenced by the combined XRD and solid‐state NMR results. Increasing the illumination time does not affect the PL peak position, indicating good phase stability of the CdI_2_ film (Figure S13, Supporting Information) with no halide demixing or photodarkening/photobrightening effects.^[^ ^14^, ^52^ ^]^ Time‐resolved PL (TRPL) spectra in Figure 5b show a bi‐exponential decay for both perovskite films, suggesting a combination of bulk and surface charge recombination process, whose lifetimes are denoted as *τ*_1_ and *τ*_2_, respectively. The fitted data in Table S5 (Supporting Information) show that *τ*_1_ and *τ*_2_ for the CdI_2_‐doped film are longer than those for the reference sample, indicating that non‐radiative charge recombination has been reduced in the CdI_2_‐doped film. We also found a remarkable increase in the average charge carrier lifetime of 79.2 ns upon CdI_2_ doping, almost three times longer than in the reference film. This result can be rationalized considering the cross‐sectional SEM images which show that in the direction normal to the film there is only a single grain throughout the film thickness, consistent with the support for fast charge transport (Figure S14, Supporting Information). The lifetimes of perovskite films coated on TiO_2_ substrate do not show apparent difference, indicating that the slight upward shift of conduction band induced by Cd‐incorporation observed in UPS does not hamper the interfacial electron injection (Figure S15 and Table S6, Supporting Information).

Femtosecond transient absorption spectroscopy (F‐TAS) measurements were carried out to further investigate the carrier recombination and transfer kinetics in the films. The decay dynamics in reference and CdI_2_‐doped films was probed using 680 nm pump light with an excitation density of <2 µJ cm^−2^ to avoid extensive Auger recombination. Figure 5c,d shows the visual photoinduced absorption as a function of the delay time and wavelength. The recovery kinetics of the bandgap bleach signal was extracted from the TAS plot in Figure 5e. Upon excitation, the negative ground state bleaching (GSB) band is centered at 701 nm. The decay dynamics follows a bi‐exponential behavior, with average time constants of 78.8 and 16.3 ns for CdI_2_‐doped film and reference film, respectively. The slower decay of the CdI_2_‐doped film compared to the reference film reflects lower recombination rate, which we attribute to the presence of fewer trap states to capture/scatter the carriers in the former film. In the case of a film including the spiro‐OMeTAD hole transport layer (HTL) (Figure 5e and Figure S16, Supporting Information), the fitted curve of CdI_2_‐doped film shows faster decay (average lifetime of 1.3 ns) compared to that of the reference film (average lifetime of 2.0 ns). This result suggests that the CdI_2_‐doped film can transfer holes from perovskite to HTL in a more efficient way, likely because of the decrease of interfacial defect density as well as the overlap of the work functions as is evident from the UPS analysis.

The space charge limited current (SCLC) method was further used to quantify the defect density (*N*_trap_), as displayed in Figure 5f,g, using FTO/TiO_2_/perovskite/[6,6]‐phenyl‐C61‐butyric acid methyl ester (PCBM)/Ag as the device architecture. The linear region at low bias voltages reveals an Ohmic‐type feature, and a marked increase of the current injection follows the increase of the bias voltage at the intermediate region, which we identify as the trap‐filling process. The kink point between these two regions is defined as the trap‐filling limit voltage (*V*_TFL_), and therefore the trap density (*N*_trap_) can be calculated from the following equation

(1) $$V_{\mathrm{TFL}}=eN_{\mathrm{trap}}L^{2}/2\varepsilon \varepsilon_{0}$$

where *e* is the elementary charge, *ε* is the relative dielectric constant, *ε*_0_ is the vacuum permittivity, and *L* is the thickness of the perovskite film. The corresponding *N*_trap_ values are calculated to be 6.84 × 10^15^ and 4.16 × 10^15^ cm^−3^ for the reference and CdI_2_‐doped devices, respectively, indicative of reduced trap density upon CdI_2_ doping. Furthermore, the capacitance–voltage (*C*–*V*) measurement was performed to probe the built‐in potential (*V*_bi_) (Figure 5h). The *V*_bi_ value determined from the intercept of the Mott–Schottky plots at 10 kHz with the *x*‐axis is 1.10 V for the CdI_2_‐doped film, which is 130 mV greater than that for the reference film. The higher *V*_bi_ is beneficial for the separation of photogenerated electron–hole pairs and charge transport to the respective charge collecting layers, facilitating fast charge collection and less carrier accumulation within the device. We also measured the *V*_OC_ of the PSCs made with and without CdI_2_ doping under different light intensities. We found a steeper increase in *V*_OC_ as a function of the light intensity in the CdI_2_‐doped device compared to the reference device (Figure 5i). The deviation of the slope from unity *kT*/*q* suggests trap‐assisted recombination, where *k* is Boltzmann constant, *T* is the absolute temperature, and *q* is the elementary charge. The CdI_2_‐doped PSCs show a slope of 1.44 *kT*/*q*, which is much smaller than that in the reference device (2.21 *kT*/*q*). This result indicates a substantially reduced trap‐assisted recombination resulting from suppressed recombination. We attribute the origin of this result to the high quality of the perovskite film achieved thanks to CdI_2_ doping, the strong electrostatic interaction between Cd^2+^ and I^−^, and the additional supply of iodide from CdI_2_. The combination of these effects compensates for the loss of iodide anions and prevents them from migrating, thus limiting the formation of new iodide vacancies. It should be noted that compared to the reported *V*_OC_ improvement for inorganic PSCs,^[^ ^13^, ^53^, ^54^ ^]^ the suppressed defect in our work results in a moderate improvement of *V*_OC_ by 30 mV (see Table 1), which is probably counter‐balanced by the decrease of perovskite bandgap after Cd^2+^‐doping.

Next, we evaluated the stability of the perovskite films and PSCs with and without CdI_2_ doping. The films were prepared and stored in an environment with a relative humidity (RH) between 40% and 50%, and snapshots of these films were taken periodically (Figure S17, Supporting Information). The reference film maintained its black phase for less than 24 h before the appearance of yellow dots. It then completely transformed into the yellow phase in less than 48 h. The CdI_2_‐doped films retained the black phase for substantially longer time than the reference film, although they too become yellow within 48–72 h. Notably, the film with 8 mol% CdI_2_ doping demonstrated the best stability by preserving the black phase for nearly 72 h. This result strongly suggests that CdI_2_ doping can substantially improve the black phase stability of iodide‐rich inorganic perovskites. Previous studies have shown that the black phase of inorganic halide perovskites with low bandgaps suffers from the transition to the yellow phase in ambient atmosphere due to the unfavorable tolerance factor that causes severe lattice distortion.^[^ ^55^, ^56^ ^]^ CdI_2_ doping may alleviate such lattice distortion by releasing the lattice strains, thus improving ambient stability. Moreover, this strategy is also effective at reducing the defect density within the film, minimizing the possibility of moisture–surface interactions which have been shown to result in vacancy‐mediated material decomposition.^[^ ^19^ ^]^ The unencapsulated inorganic PSCs were also stored in an ambient environment with a RH of 20% for stability evaluation (Figure S18, Supporting Information). PSCs with CdI_2_ doping exhibit a mild decrease of the PCE within 600 h and retain 90% value of the initial efficiency at the end of the test. On the contrary, the PCE for the reference PSCs drops to ≈80% of the initial value, indicating that the introduction of CdI_2_ is beneficial for improving the ambient stability of inorganic PSCs. Finally, we note that while cadmium is a toxic heavy metal with the potential for negative environmental impact, in our work we explore fundamental ways of improving the performance and stability of solar cells to constructively contribute to the global energy challenge. We hope that developing an understanding of these, admittedly toxic, but highly performant solar cell materials will pave the way to the discovery of more environmentally friendly alternatives based on similar underlying atomic‐level principles.

## 3 Conclusions

In conclusion, we have introduced CdI_2_ as an additive into CsPbI_3‐x_Br_x_ and used the resulting material as an active layer to fabricate high‐performance inorganic PSCs. Three functions were revealed upon the addition of CdI_2_. First, Cd^2+^, which has a smaller radius than Pb^2+^, partially replaces Pb^2+^ inside the CsPbI_3−_ *_x_* Br*_x_* perovskite lattice, with the substitution threshold of ≈2 mol%. This results in a substantially increased DFE for halide vacancies, which we attribute to reduced lattice distortion of the [PbX_6_]4^−^ octahedral cages. Second, for doping level beyond 2 mol%, the excess Cd^2+^ forms Cd‐rich phases of the type Cs_2_CdI_4−_ *_x_* Br*_x_* (*x* ≈ 0.3), which remain on the surface of the film and at GBs and reduce nonradiative recombination. Third, the extra iodide introduced with CdI_2_ is also incorporated into the perovskite lattice, filling iodide vacancies and compensating for the iodide loss. The combination of these three functions results in increased charge carrier lifetimes, decreased GB surface area, as well as an upward shift of the perovskite VB in the CdI_2_ doped films. The *V*_OC_, *J*_SC_, and FF were enhanced with CdI_2_ doping, yielding devices with a champion PCE of 20.8%, one of the highest values reported to date for inorganic PSCs. The ambient stabilities of the films and devices were also improved with CdI_2_ doping. Our work suggests that both lattice engineering and defect control are important in achieving high‐performance inorganic PSCs and the CdI_2_ additive engineering is promising for performance enhancement for iodide‐rich inorganic PSCs. We also show that the combination of long‐range (XRD) and local structure (solid‐state NMR) characterization techniques with comprehensive optoelectronic characterization is the most productive approach to understanding structure–performance relationships of dopants in inorganic halide perovskites.

## 4 Experimental Section

### Materials

FTO glass was purchased from Asahi Glass Company (AGC), Japan. Cesium iodide (CsI, 99.999%), DMAPbI_3_, lead bromide (PbBr_2_, 99.99%), lead iodide (PbI_2_, 99.5%), PCBM, and Spiro‐OMeTAD were purchased from Xi'an Polymer Light Technology. Cadmium iodide (CdI_2_, 99.9985%), N, N‐dimethylformamide (DMF, ≥99.8%), dimethyl sulfoxide (DMSO, ≥99.9%), lithium bis‐(trifluoromethanesulfonyl) imide (LiTFSI), and 4‐*tert*‐butylpyridine (*t*BP, 96%) were purchased from Thermo Fisher Scientific. Titanium tetrachloride (TiCl_4_,≥98%) and chlorobenzene (CB) were obtained from China National Pharmaceutical Group Corporation. All other chemicals were obtained from Sigma Aldrich.

### Device Fabrication

The FTO glass (2.5 × 2.5 cm^2^) was cleaned sequentially with acetone, isopropanol, and ethanol for a total time of 30 min in an ultrasonic bath. The cleaned FTO glass was surface‐treated by plasma for ≈10 min and then immersed into 40 × 10^−3^ m TiCl_4_ solution at 70 °C for 1 h to deposit an ≈50 nm TiO_2_ compact layer (*c*‐TiO_2_). These TiO_2_ substrates were annealed at 200 °C for 30 min and then immediately transferred into a N_2_‐filled glovebox. 0.6 m perovskite precursor solutions were prepared by mixing CsI, DMAPbI_3_, and PbBr_2_ with a molar ratio of 3.0:2.8:0.2 in a mixture solvent of DMSO and DMF (1:4, v/v). Different amounts of CdI_2_ (3, 8, and 13 mol% relative to Pb) were added to prepare Cd‐containing precursor solutions. These solutions were stirred at room temperature for 12 h for complete dissolution and were used after filtering through a syringe filter with a 0.45 µm pore size. The perovskite films were spin‐coated on the TiO_2_ substrates at 4000 revolutions per minute (rpm) for 40 s and then annealed at 210 °C for 5 min. For the HTL solution, 90 mg spiro‐OMeTAD was dissolved in 1 mL CB with 36 µL *t*BP and 22 µL LiTFSI (520 mg LiTFSI was dissolved in 1 mL acetonitrile). The solution was stirred at room temperature for 12 h. The HTL solution was spin‐coated on perovskite films at 5000 rpm for 30 s. A 80 nm thick Au metal contact was deposited on the top of the spiro‐OMeTAD layer by thermal evaporation to complete the device.

### Solid‐State Mechanosynthesis

The materials used for solid‐state NMR characterization were prepared using mechanosynthesis following the previously published protocol.^[^ ^40^, ^41^ ^]^ In brief, the precursors were stored under argon and weighed out into an agate grinding jar (10 mL) containing an agate ball (⌀10 mm). The precursors were ground in an electric ball mill (Retsch MM−400) for 30 min at a vibration frequency of 25 Hz. The resulting powders were scraped off the walls of the grinding jars, transferred into glass vials and annealed for 5 min at 300 °C to remove grinding‐induced defects and in the case of CsPb_0.92_Cd_0.08_I_2.8_Br_0.2_ and CsPbI_2.8_Br_0.2_ induce the phase transition to the perovskite phase.

The following amounts of precursors were used:

CsPb_0.92_Cd_0.08_I_2.8_Br_0.2_: CsI (104 mg, 0.40 mmol), CsBr (21 mg, 0.1 mmol), CdI_2_ (15 mg, 0.04 mmol), and PbI_2_ (212 mg, 0.46 mmol);

CsPbI_2.8_Br_0.2_: CsI (104 mg, 0.40 mmol), CsBr (21 mg, 0.1 mmol), and PbI_2_ (231 mg, 0.5 mmol); and

Cs_2_CdI_3.73_Br_0.27_: CsI (225 mg, 0.865 mmol), CsBr (29 mg, 0.135 mmol), and CdI_2_ (183 mg, 0.50 mmol).

### Characterizations

XRD patterns of the samples were measured using a D/MAX 2400 Diffractometer with Cu K*α* radiation (1.5405 Å) (DX‐2400). The XPS and UPS measurements were performed on a VG ESCALAB MK2 system with monochromatized Al K*α* radiation. The depth‐profiling XPS used an Ar ion beam with a sputter rate of 0.48 nm s^−1^. UV–vis absorption spectra were acquired using a Shimadzu UV‐3600 spectrofluorometer. The top‐view and cross‐sectional SEM images of the perovskite films and solar cells were performed using a field‐emission scanning electron microscope (HITACHI SU‐8020). The AFM images were acquired using a Bruker Dimension Icon (Bruker Nano, Inc.). The PL (excitation at 510 nm) and TRPL (excitation at 510 nm) spectra were obtained using a PicoQuant FluoTime 300 fluorescence spectrometer. F‐TAS measurements were performed utilizing a commercial TA system (Time‐Tech Spectra, LLC) equipped with a high‐speed spectrometer (Ultrafast systems, HELIOS) and a regeneratively amplified Ti:Sapphire laser (light conversion, 1030 nm, 150 fs, and 100 kHz repetition) with 680 nm wavelength and 100 kHz repetition rate (Coherence) and served as both pump and probe beams. The DLS of the perovskite precursor solutions was performed using a Brookhaven 90Plus particle size analyzer. HAADF‐TEM images of perovskite crystals and elemental maps were obtained using a field‐emission transmission electron microscope (JEOL, JEM‐2800). DFT calculations were performed using the Vienna Ab initio Simulation Package (VASP) within the framework of the generalized gradient approximation (GGA) with the Perdew–Burke–Ernzerhof (PBE) functional. Projected augmented wave (PAW) potentials were used to show the ionic cores and take valence electrons into account using a plane wave basis set with a kinetic energy cutoff of 400 eV. The relaxation of bulk and surface slabs were performed with the force convergence less than 0.05 eV Å^−1^.

Solid‐state MAS NMR spectra of ^113^Cd (110.99 MHz) and ^133^Cs (65.59 MHz) were recorded on a Bruker Avance III 11.7 T spectrometer with a 4 mm probe. ^133^Cs shifts were referenced to a 1 m aqueous solution of cesium chloride, using solid CsI (*δ* = 271.05 ppm) as a secondary reference.^[^ ^57^ ^] 113^Cd spectra were acquired using an echo sequence to eliminate distortions due to receiver dead time. The refocusing pulse was either a hard *π* or a tanh/tan pulse (as specified in Tables S1 and S2, Supporting Information), with the latter being used to ensure that all species are detected in case the ^113^Cd chemical shift range is too large to be covered by a single hard *π* pulse. The tanh/tan pulse had a duration of 80 µs and a bandwidth of 1 MHz. For the saturation‐recovery experiments, saturation was achieved by applying a train of 15 *π*/2 pulses spaced by 15 ms. About 200 mg of material was used for each measurement, corresponding to a full 4 mm zirconia rotor. The spectra were fitted using Mestrenova (Mestrelab).

The current–voltage (*J–V*) curves of the PSCs were measured on a solar simulator (SS‐F5‐3A, Enlitech) emitting an approximate AM 1.5 G spectrum whose intensity was calibrated at 100 mW cm^−2^ using a certified standard silicon solar cell (SRC‐2020, Enlitech). Device active area was defined by a mask with an aperture area of 0.09 cm.^[^ ^2^ ^]^ The IPCE was measured using the QTest Station 2000 ADI system (Crowntech, Inc.). SCLC was acquired by the solar cells in the dark environment to monitor *J–V* characteristics and measure the defect density. The electron‐only device with a configuration of FTO/*c*‐TiO_2_/perovskite/PCBM/Ag was prepared. The 20 mg PCBM in 1 mL CB solution was deposited on perovskite layer at 2000 rpm for 30 s. A 120 nm thick Ag metal was then deposited by thermal evaporation. The measurement was performed with a scan rate of 0.02 V in a dark environment at room temperature. The *C–V* measurement was performed using a Zahner Zennium electrochemical workstation. The *V*_OC_ dependence on the light intensities was acquired by *J–V* setup. The stability tests were carried out by storing film and devices in an ambient environment with RH of 40%–50%. The PCE evolution of the devices was obtained through periodical *J–V* measurement.

## Conflict of Interest

The authors declare no conflict of interest.

## Supporting information

Supporting Information

## Data Availability

The data that support the findings of this study are available from the corresponding author upon reasonable request.
