# Supplementary material for: Simultaneous Lattice Engineering and Defect Control via Cadmium Incorporation for High‐Performance Inorganic Perovskite Solar Cells
Source: Adv Sci (Weinh). 2022 Nov 7;9(36):2204486. doi: 10.1002/advs.202204486 (PMC9798970; doi:10.1002/advs.202204486)
Supplement: Supplementary file 1 — Supporting Information [file ADVS-9-2204486-s001.pdf]

ADVANCED SCIENCE
Open Access

Supporting Information

for *Adv. Sci.*, DOI 10.1002/advs.202204486

Simultaneous Lattice Engineering and Defect Control via Cadmium Incorporation for High-Performance Inorganic Perovskite Solar Cells

Tianfei Xu, Wanchun Xiang, Dominik J. Kubicki, Yali Liu, Wolfgang Tress and Shengzhong Liu**

Supporting information for

Simultaneous lattice engineering and defect control via cadmium incorporation for high-performance inorganic perovskite solar cells

Tianfei Xu[1], Wanchun Xiang[1,*], Dominik Kubicki[2], Yali Liu[1], Wolfgang Tress[3], Shengzhong Liu[1,4,*]

[1]Key Laboratory of Applied Surface and Colloid Chemistry, Ministry of Education, Shaanxi Key Laboratory for Advanced Energy Devices, Shaanxi Engineering Lab for Advanced Energy Technology, School of Materials Science and Engineering, Shaanxi Normal University, Xi'an, 710119, China. wanchun.xiang@snnu.edu.cn

[2]Department of Physics, University of Warwick, Coventry, CV4 7AL, UK

[3]Institute of Computational Physics, Zurich University of Applied Sciences, Wildbachstr. 21, 8401 Winterthur, Switzerland

[4]Dalian National Laboratory for Clean Energy; iChEM, Dalian Institute of Chemical Physics, Chinese Academy of Sciences, Dalian 116023, China. E-mail: szliu@dicp.ac.cn

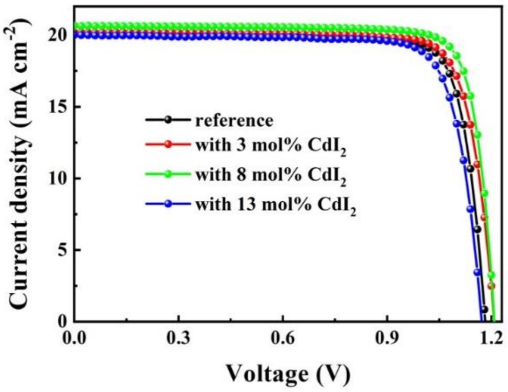

Figure S1. *J-V* curves of $CsPbI_{3-x}Br_x$ inorganic perovskite solar cells with different concentration of Cd-doping. The measurement was performed under one sun irradiation with an aperture area of 0.09 mm^2.

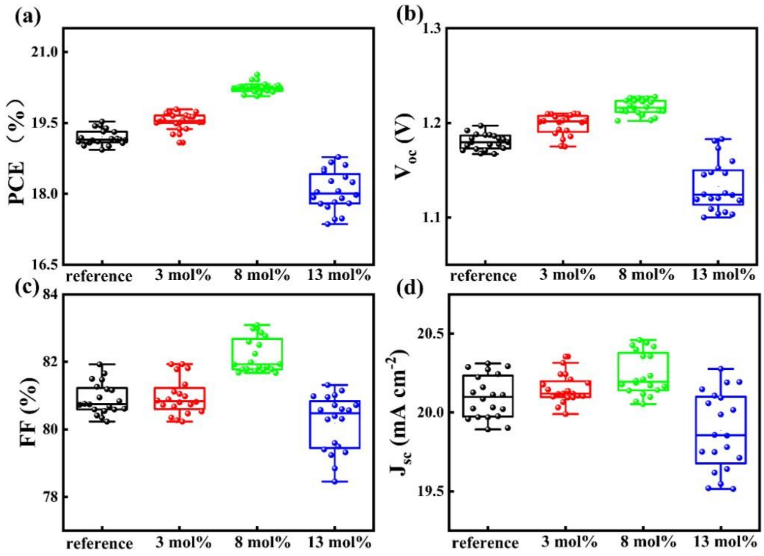

Figure S2. Statistics of photovoltaic performance for $CsPbI_{3-x}Br_x$ inorganic PSCs with different concentration of Cd-doping. (a) PCE; (b) V_{OC}; (c) FF; (d) J_{SC}.

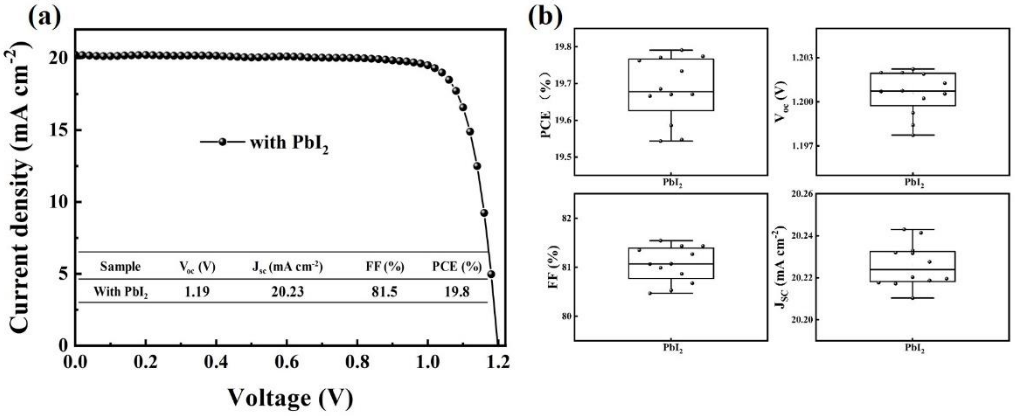

Figure S3. (a) Photovoltaic performance for $CsPbI_{3-x}Br_x$ inorganic perovskite solar cells with 8 mol% PbI_2 as additive. The measurement was performed under one sun irradiation with an aperture area of 0.09 cm^2. (b) The statistics of photovoltaic performance of PbI_2-containing PSCs.

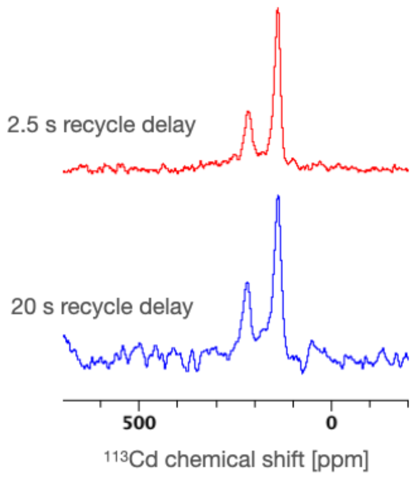

Figure S4. ^{113}Cd MAS NMR spectra of $CsPb_{0.92}Cd_{0.08}I_{2.8}Br_{0.2}$ made by solid-state mechanosynthesis, recorded with a shorter (2.5 s; 32768 scans, 22.8 h total acquisition time) and longer (20 s; 2340 scans, 13 h total acquisition time) recycle delay.

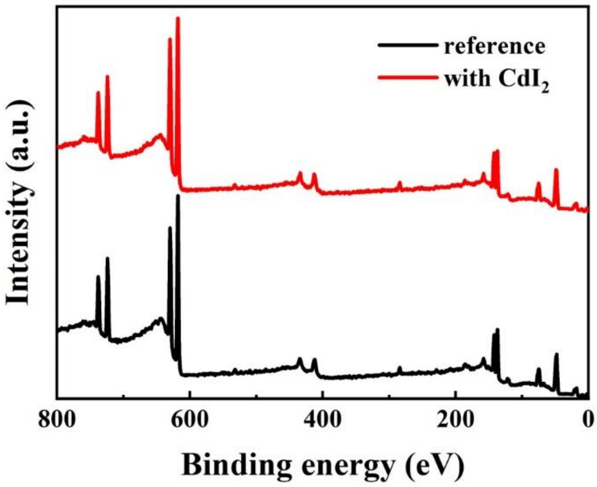

Figure S5. XPS full scan of the $CsPbI_{3-x}Br_x$ inorganic perovskite films without and with CdI_2.

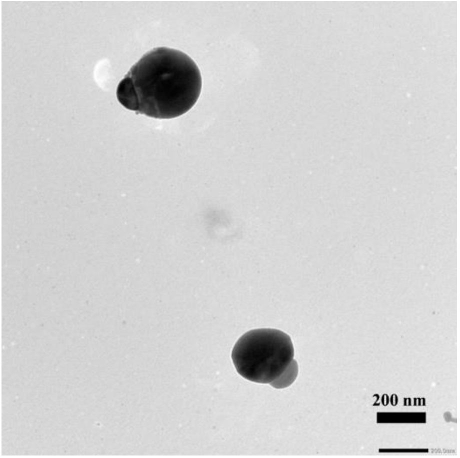

Figure S6. TEM image of $CsPbI_{3-x}Br_x$ perovskite sample with Cd-doping. The scale bar is 200 nm.

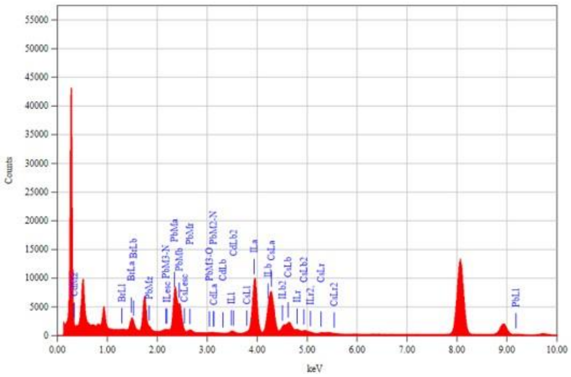

Figure S7. Energy-dispersive X-ray (EDS in a TEM) elemental mapping of $CsPbI_{3-x}Br_x$ perovskite sample with 8 mol% Cd-doping.

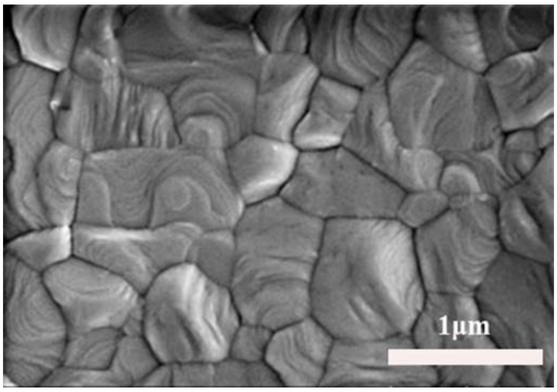

Figure S8. Top-view SEM image of $CsPbI_{3-x}Br_x$ perovskite film with 8 mol% PbI_2 addition. The scale bar is 1 μm.

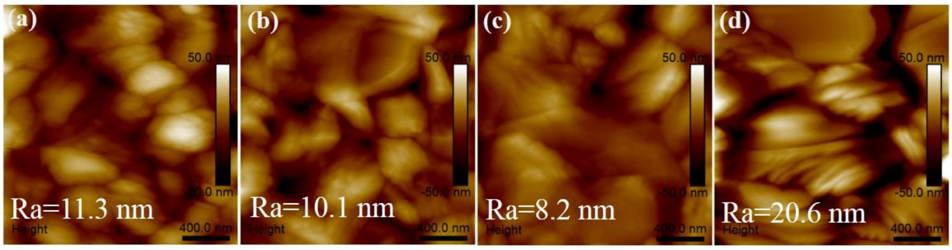

Figure S9. AFM characterization of $CsPbI_{3-x}Br_x$ perovskite film with different concentration of CdI_2. The scale bar is 400 nm. Ra represents root mean square (RMS). (a-d): reference; 3 mol%; 8 mol%; 13 mol%.

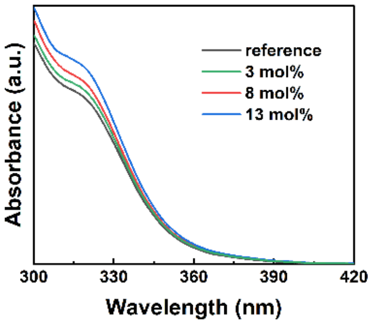

Figure S10 UV-vis spectra of perovskite precursor solution with different concentrations of CdI_2.

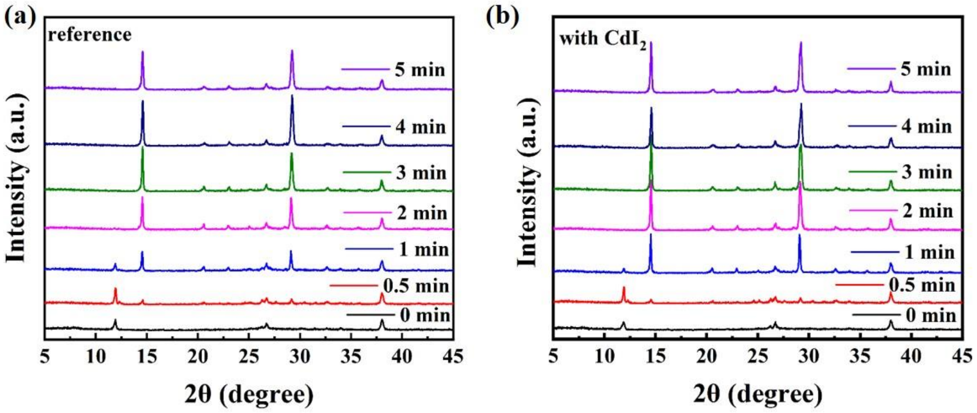

Figure S11. In-situ XRD characterization of $CsPbI_{3-x}Br_x$ perovskite film with different annealing time. (a) reference, (b) with 8 mol% CdI_2.

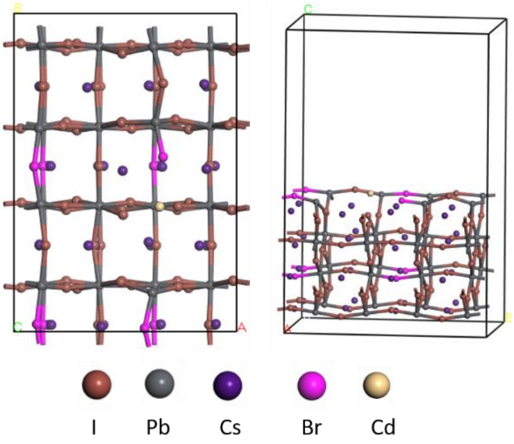

Figure S12. Top view of the iodide vacancy defects upon Cd-doping in (100) CsPbCdIBr-slab using DFT calculation.

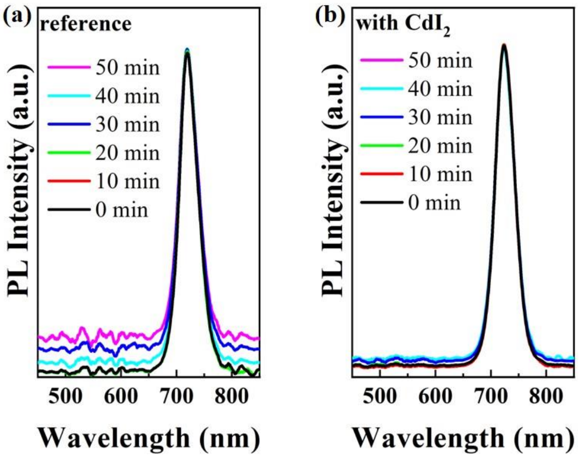

Figure S13. Time dependence of PL curves of perovskite films (a) reference; (b) with 8 mol% CdI_2.

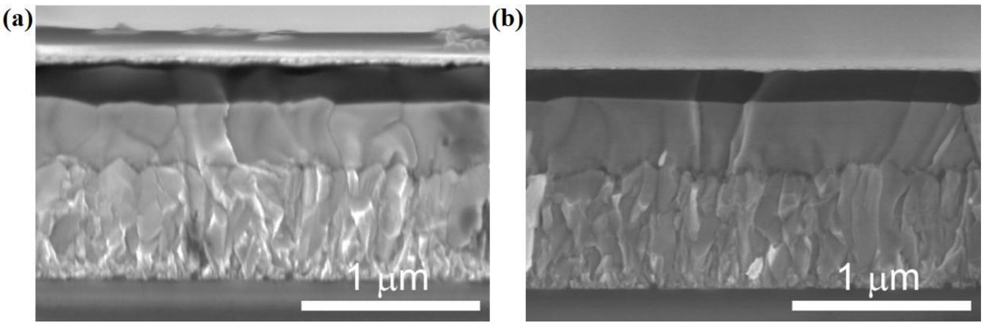

Figure S14. Cross-sectional SEM images of inorganic PSCs (a) reference; (b) with Cd-doping. The scale bar is 1 μm.

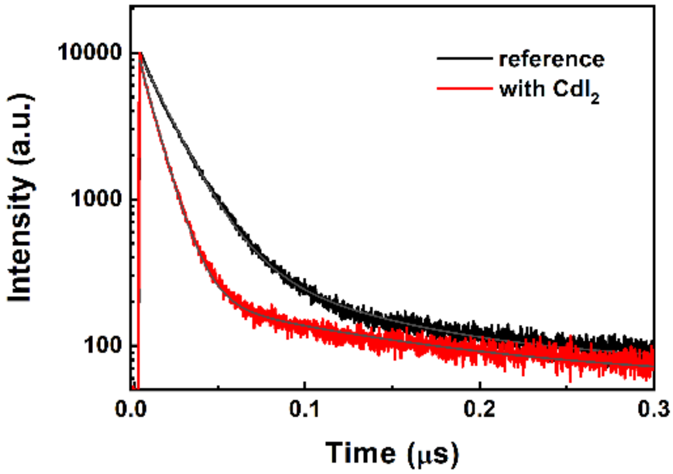

Figure S15 TRPL curves of perovskite with and without CdI_2 addition. The films were prepared on TiO_2 substrate.

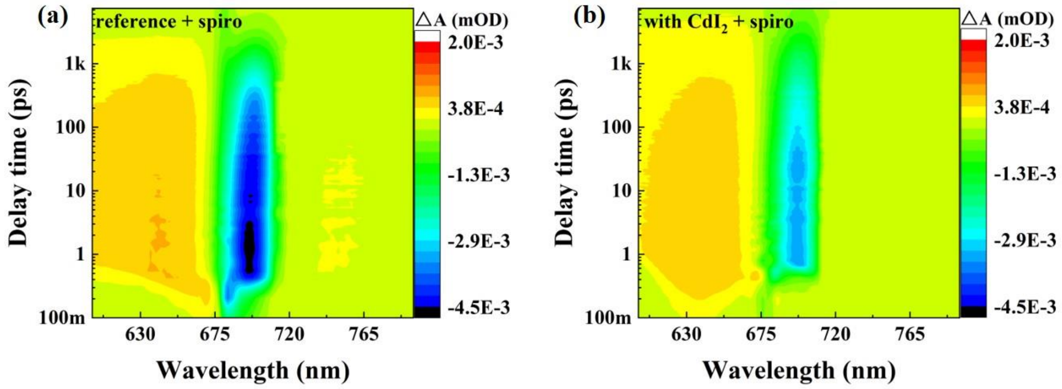

Figure S16. 2D contour plot of TAS of the photoinduced absorption (ΔA) as a function of wavelength and delay time for films a) reference; b) with Cd-doping. Both perovskite films were capped with spiro-OMeTAD hole transport layer.

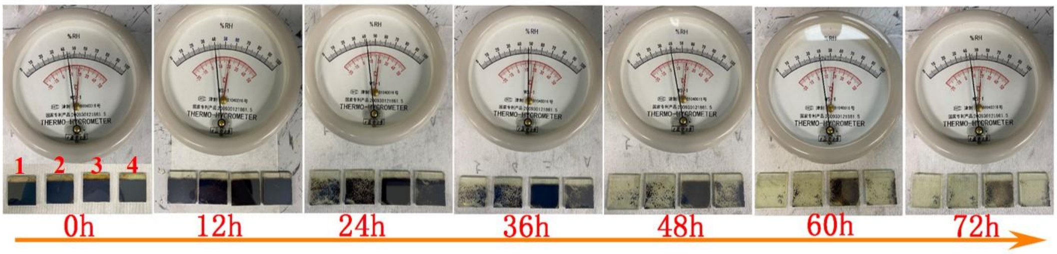

Figure S17. Stability of $CsPbI_{3-x}Br_x$ perovskite films with different CdI_2 concentrations. These films were stored in an environment with a RH between 40% and 50%. (1: reference; 2: 3 mol%; 3: 8 mol%; 4: 13 mol%)

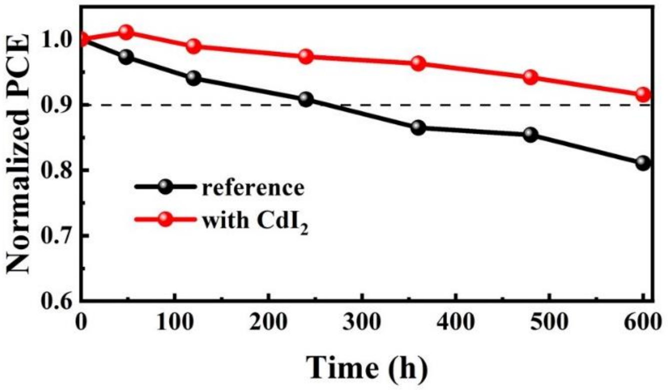

Figure S18. Device stability without and with 8 mol% CdI_2 addition. The devices were stored in an ambient environment with a RH of 20%.

Table S1. ^{113}Cd NMR acquisition and processing parameters for the materials shown in Figures 2d and S4. Magnetic field strength: 11.7 T, MAS spin rate: 12.5 kHz.

Material	Pulse sequence	Number of scans	Recycle delay (s)	Total experiment time (h)	Apodization (Hz)
$CsPb_{0.92}Cd_{0.08}I_{2.8}Br_{0.2}$	Hahn echo	32,768	2.5	22.8	1000
$CsPb_{0.92}Cd_{0.08}I_{2.8}Br_{0.2}$	Echo with tanh/tan refocusing	2,654	20	14.7	1000
$Cs_2CdI_{3.73}Br_{0.27}$	Echo with tanh/tan refocusing	2,695	2.5	1.9	500

Table S2. ^{133}Cs NMR acquisition and processing parameters for $CsPb_{0.92}Cd_{0.08}I_{2.8}Br_{0.2}$ in Figures 2d and 2e. Magnetic field strength: 11.7 T, MAS spin rate: 12.5 kHz.

Material	Pulse sequence	Number of scans	Recycle delay (s)	Total experiment time (h)	Apodization (Hz)
$CsPb_{0.92}Cd_{0.08}I_{2.8}Br_{0.2}$	Hahn echo	4	600	0.7	100
$CsPbI_{2.8}Br_{0.2}$	Hahn echo	8	600	1.3	100
$Cs_2CdI_{3.73}Br_{0.27}$	Hahn echo	4	300	0.3	50

Table S3 Hysteresis behavior of PSCs with and without CdI_2. The measurement was carried out under 100 mW cm^{-2} light intensity with a scan rate of 30 mV s $^{-1}$. The aperture area is 0.09 cm^2.

Sample	V_{oc} (V)	J_{sc} (mA cm^{-2})	FF (%)	PCE (%)
reference-reverse	1.18	20.14	81.9	19.4
reference -forward	1.16	20.14	80.1	18.7
with CdI_2-reverse	1.21	20.64	83.2	20.8
with CdI_2-forward	1.19	20.68	82.9	20.3

Table S4 The optical properties of perovskite thin films with and without CdI_2 addition by UPS characterization.

	$E_{cut\text{-}off}$ (eV)	E_V-E_F (eV)	E_F (eV)	E_V (eV)	E_C (eV)	E_g (eV)
reference	17.28	1.34	3.94	5.28	3.53	1.75
with CdI_2	17.48	1.43	3.74	5.17	3.43	1.74

Table S5 Charge lifetimes within perovskite films with and without CdI_2 addition from TRPL measurement.

	A_1 (%)	T_1 (ns)	A_1 (%)	T_1 (ns)	T_{ave} (ns)
reference	17.0	46.8	82.9	14.3	27.4

with CdI_2	19.1	124.2	80.8	23.8	79.2

Table S6 Charge lifetimes of perovskite films with and without CdI_2 addition from TRPL measurement. The films were prepared on TiO_2 substrate.

	**A_1 (%)**	**T_1 (ns)**	**A_1 (%)**	**T_1 (ns)**	**T_{ave} (ns)**
reference	13.56	32.5	86.44	20.3	21.8
with CdI_2	18.01	28.4	81.99	19.7	22.7
